# Layer-selective magnetization switching in the chirped photonic crystal with GdFeCo

**DOI:** 10.1038/s41598-021-81887-0

**Published:** 2021-01-26

**Authors:** O. V. Borovkova, D. O. Ignatyeva, V. I. Belotelov

**Affiliations:** 1 Faculty of Physics, Lomonosov Moscow State University, Moscow, Russia; 2 Russian Quantum Center, Skolkovo, Russia

**Keywords:** Nanophotonics and plasmonics, Magneto-optics

## Abstract

Here we propose a magnetophotonic structure for the layer-selective magnetization switching with femtosecond laser pulses of different wavelengths. It is based on a chirped magnetophotonic crystal (MPC) containing magnetic GdFeCo and nonmagnetic dielectric layers. At each operating wavelength the laser pulses heat up to necessary level only one GdFeCo layer that leads to its magnetization reversal without any impact on the magnetization of the other layers. Moreover, magneto-optical reading of the MPC magnetization state is discussed. Lateral dimensions of the considered MPC can be made small enough to operate as a unit cell for data storage.

## Introduction

Up-to-date technologies require modern information devices to process large amounts of data at high rates. Methods of the ultrafast magnetism are very promising in this respect since they allow controlling the magnetization of a magnetic material by the femtosecond laser pulses at a picosecond time scales^1–10^.

Ferrimagnetic alloy of gadolinium-iron-cobalt (GdFeCo) is one of the most promising materials for further development of the information storage devices, since it allows to perform a single-shot all-optical magnetization switching during a few picoseconds^11–13^ in the area of pulse action on the GdFeCo film. The magnetization state of the remagnetized area determines the value of the recorded information bit, one direction for ‘0’, and another direction for ‘1’. A mechanism of all-optical switching originates from the ultrafast spin dynamics of the exchange-coupled Gd and FeCo sublattices which are thrown out of balance due to the light-induced thermal heating of the spin sublattices^14,15^. If the delivered energy exceeds the threshold, the magnetizations of the two sublattices are reversed at the picosecond timescales due to the difference of the relaxation times for these sublattices^16^. Thus, ultrafast all-optical magnetization switching depends on the amount of energy delivered by the optical pulse^17^ and is characterized by a threshold femtosecond pulse intensity required for the magnetization switching^18,19^.

Nowadays many efforts are put to study how to control the process of all-optical switching via tailoring the properties of the femtosecond pulse^19–21^ and via the additional patterning of the magnetic structure and surface plasmon excitation^22–25^. The latter is responsible for strong light localization^24–26^ which enables layer-selective all-optical switching in a bilayer heterostructure of GdFeCo that has been demonstrated recently^24^. However, the disadvantage of the SPP-assisted selectivity^24^ is that it intrinsically requires large amounts of metal to support SPP which cause about 20% non-resonant absorption in each layer. Consequently, plasmonic-based approach could not be extended to multilayered structures with large number of GdFeCo layers in a multilayer stack. At the same time, recent studies showed the advantage of all-dielectric 1D and 2D periodic magnetic nanostructures for local and efficient light-spin interaction^10,27,28^.

In this work, we propose a design of nanostructure where the parameters of the dielectric rather than metal layers determine the spatial light distribution and the resonances of the absorption in the GdFeCo layers. We propose to use the photonic crystals, which are all-dielectric structures that allow to reconfigure the electromagnetic field distribution by the frequency detuning^29,30^. The magnetophotonic crystal (MPC) structures^31–34^, i.e., the multilayer nanostructures containing the magnetic layers were shown to perform tunable localization of light at the resonant frequencies enabling the enhancement of the optomagnetic interaction in a magnetic material^34^.

The proposed magnetophotonic crystal with thin smooth or patterned layers of GdFeCo ferrimagnetic alloy that provides the local magnetization control and targeted remagnetization of the single layers of the multilayer structure. By the frequency tuning of the input femtosecond laser pulses one can select the magnetic layer of GdFeCo in which the energy of the laser pulse is mostly concentrated and exceeds the magnetization reversal threshold, while in others it is 1.5–2 times lower. In other words, the proposed MPC allows to ‘turn on’ one certain magnetic layer of the structure and leave all other layers ‘turned off’.

As GdFeCo material is perspective for data storage applications allowing a significant increase of the writing speed, the proposed structure can be implemented for the purposes of the record density increase in such a device. Usually, the magnetic bit cells are arranged in the plane of a single magnetic layer. Using the proposed MPC, bit cells can be arranged in several magnetic layers separated by the submicron dielectric spacers and addressed individually. This configuration is quite simple for fabrication and doesn’t require a precise positioning of the laser pulse.

Besides that, another configuration of the chirped MPC structure with the patterned GdFeCo layers is addressed. For the higher density of the information recording the magnetic layer can be nanostructured to separate bit cells in the lateral direction. The patterning of the magnetic layer can be implemented along both axes in the plane of the structure layers. In our work we limited ourselves by one-dimensional patterning for the sake of the modelling simplicity. It should be stressed that the patterned chirped MPC allows combining two approaches instantaneously, the bit cells arranged in plane and the bit cells arranged in different layers. It opens up the ample opportunities for the significant increase of the information recording density.

We also provide the method of an all-optical single-wavelength information reading in this multilayered structure. Thus, the designed MPC with GdFeCo layers could serve as a basis of 3D information storage devices.

## A design of the chirped magnetophotonic crystals with GdFeCo layers

We propose a SiO_2_/TiO_2_ photonic crystal with thin GdFeCo layers embedded inside each pair of the dielectric layers. The optical response of such structure is determined mainly by the parameters of the dielectric layers, including the absorption of the light in each GdFeCo layer, while the dielectric layers are considered transparent^35–37^ (for the details see Supplementary S1). We have considered the structures with thin smooth layers of GdFeCo and have shown that they possess similar optical field distributions as in the patterned GdFeCo films of the same effective thickness. The decrease of the total metal amount in each GdFeCo layer due to the patterning allows reducing the non-resonant absorption and increasing the number of GdFeCo layers that could be individually addressed.

**Figure 1:**
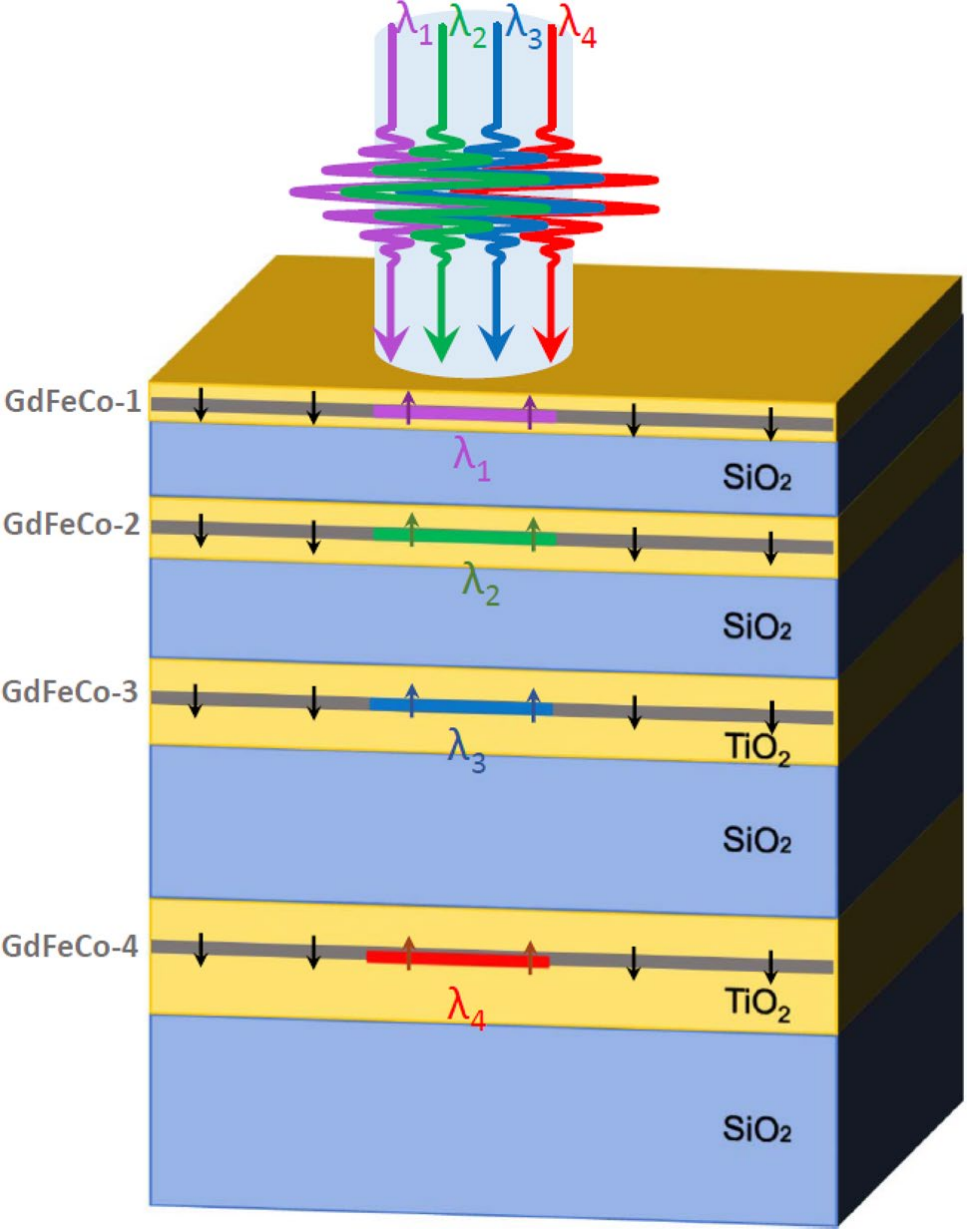
A scheme of the addressed chirped magnetophotonic crystal with thin GdFeCo layers. TiO_2_ layers (yellow) alternate with SiO_2_ layers (blue). Grey areas indicate the GdFeCo layers introduced into TiO_2_. Depending on the wavelength of the input femtosecond pulse, the different GdFeCo layers experience remagnetization due to the heating. This process is illustrated by multi-colored rectangles. For example, if the structure is illuminated at wavelength ${\lambda }_{1}$, then the energy absorbed in the GdFeCo-1 layer exceeds the threshold while it remains below the threshold for all other GdFeCo layers. As a result, only GdFeCo-1 layer is switched, while magnetization in all other layers remains untouched. The magnetization direction in GdFeCo layers is shown by small arrows. The dimensions and proportions are not respected and are chosen just for ease of perception (for the correct dimensions see Table 1).

We consider a 1D chirped MPC structure composed of the four pairs of non-magnetic layers of titanium dioxide (TiO_2_) and silicon dioxide (SiO_2_) with the thicknesses gradually increasing deeper into the MPC (see Fig. 1). The thicknesses of the TiO_2_ and SiO_2_ layers are given in the Table 1. The number of the layers was chosen for the sake of simplicity and clarity. However, it can be easily extended for the greater numbers of the layers due to the transparence of the dielectric layers and the extremely small thickness of the absorptive magnetic layers. The suitable design of the photonic crystal can provide the light concentration and magnetization state switching in the even deeper layers.

**Table 1:** The layer thicknesses of the addressed chirped magnetophotonic crystal.

Material	Pair 1		Pair 2		Pair 3		Pair 4	
	TiO_2_	SiO_2_	TiO_2_	SiO_2_	TiO_2_	SiO_2_	TiO_2_	SiO_2_
Thickness (nm)	48	270	61	292	75	317	83	330

The variation of the layer’s thickness deeper inside the chirped MPC structure leads to the shift of the bandgap corresponding to the infinite photonic crystal with the same TiO_2_/SiO_2_ layer thicknesses. Varying the frequency of the input pulses on may achieve that the light is efficiently reflected from the predefined pair of layers, while the magnitude of the electromagnetic field in the deeper layers will be significantly lower.

The energy of the EM field is mostly concentrated in the layers with higher refractive index at the long-wavelength edge of the bandgap (and vice versa). As soon as we operate at the long-wavelength edge of the bandgap due to the growth of the layer’s thickness deeper inside the MPC, we expect higher concentration of the EM field in the TiO_2_ layers having greater refractive index. Actually, the refractive index of SiO_2_ is ${n_{Si{O_2}}} = 1.45$, and for TiO_2_ layers ${n_{Ti{O_2}}} = 2.58$ at $\lambda = 644nm$ and their thickness increase for longer wavelengths^38^. Therefore, we introduce the thin layers of GdFeCo into the TiO_2_ layers to provide the highest concentration of the EM field in them. The thin magnetic layers of GdFeCo are introduced inside the TiO_2_ layers so that the total thickness of the complex TiO_2_ and GdFeCo layer does not change (remains as it is given in the Table 1). Substituting some parts of TiO_2_ by GdFeCo ultrathin layers makes just minor changes of the optical properties of the MPC, but at the same time it ensures a strong localization of light in the layers with magneto-optical response at the ‘operating’ wavelengths that is determined by the parameters of the dielectric TiO_2_/SiO_2_ layers.

We address two types of the GdFeCo layers. The first one is a smooth layer with the thickness of 3 nm (the case is presented in Fig. 1). The second type is the patterned 9 nm-thick layer. The pattern has a form of the 1D subwavelength grating in the plane of the layers with a period of 90 nm. Actually, such patterning of the magnetic layer can be implemented along both axes in the plane of the structure layers. In our work we limited ourselves by one-dimensional patterning for the sake of the modelling simplicity. However, we don’t impose any demands of continuity to another plane axis. The thickness of GdFeCo in a patterned layer is comparable to the case of a smooth film: 30 nm-wide stripes of 9-nm thick GdFeCo alternating with 60 nm-wide stripes of TiO_2_ provide effectively the 3 nm thickness of GdFeCo material averaged over a period of the structure. We show that optical properties including the absorption distributions in a smooth and patterned structures of the same effective thickness are very close.

Additionally, we have investigated the tolerance of the proposed structure to the GdFeCo thickness variation and found that the same structure at the same wavelengths actually provides the selective light localization for 1–6 nm thick GdFeCo layer (Supplementary S2).

## Selective remagnetization of the GdFeCo layers in the chirped magnetophotonic crystal

**Figure 2:**
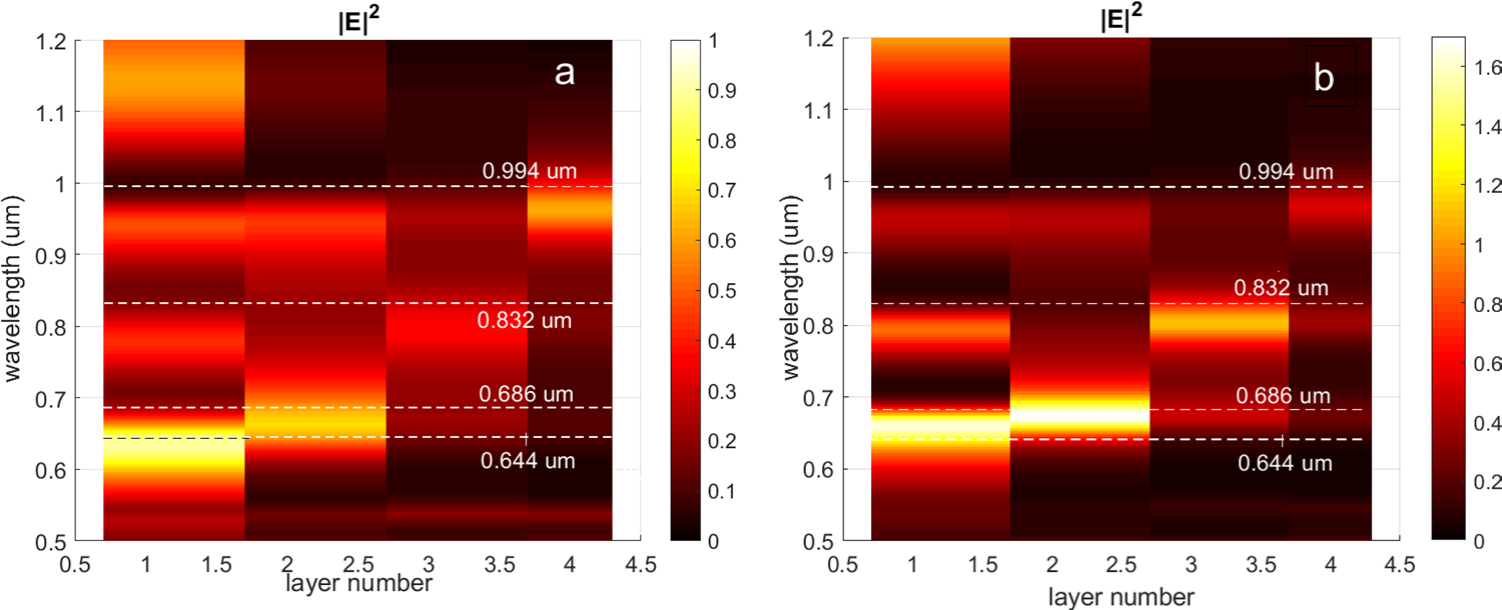
A spectral distribution of the mean |E|^2^ in TiO_2_ layers with (**a**) the smooth 3 nm-thick and (**b**) the patterned 9 nm-thick GdFeCo layers inside. Dashed lines indicate the operating wavelengths.

If the MPC is illuminated by femtosecond pulses falling normally to the MPC layers then spatial distribution of the EM field in the structure depends on the input light frequency. For the addressed MPC structure, the spectral distributions of the averaged square of the electric field absolute value, <|E|^2^ > , in the TiO_2_ layers is given in Fig. 2. The spectra are given for both smooth 3 nm-thick layer of GdFeCo (Fig. 2a) and for the 9 nm-thick patterned layer (Fig. 2b) as well. For each of four layers one can choose the wavelength when the EM energy in that layer exceeds significantly the energy in the other layers. Particularly, at the wavelengths of 644 nm, 686 nm, 832 nm, and 994 nm the light is mostly localized inside the first, second, third and fourth TiO_2_ layer of the proposed MPC, correspondingly. Both smooth and patterned GdFeCo layers provide the prevailing concentration of light in the same layer.

**Figure 3:**
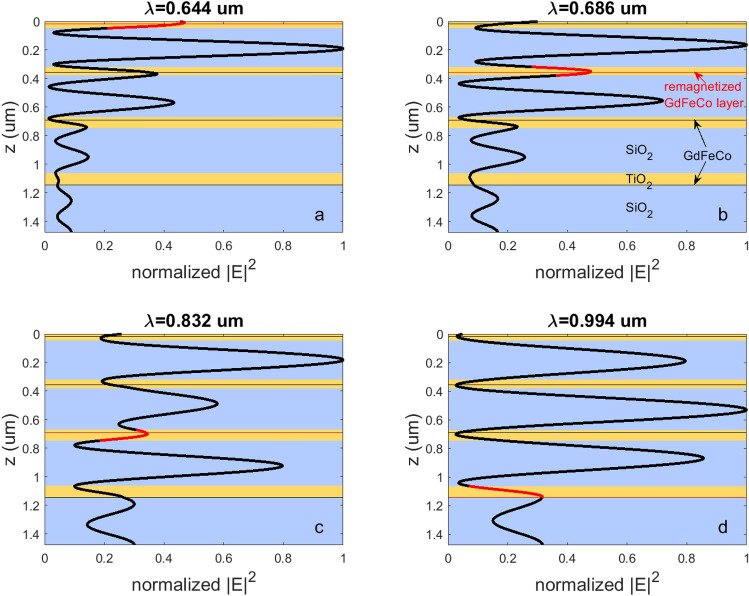
The spatial distribution of the normalized |E|^2^ in the chirped MPC with the smooth GdFeCo layer at the ‘operating’ wavelengths of (**a**) 644 nm, (**b**) 686 nm, (**c**) 832 nm, (**d**) 994 nm. Red sections of the curve show the magnitude of the normalized |E|^2^ inside the addressed GdFeCo layer and nearby. The yellow areas correspond to TiO_2_ layers, the blue regions refer to SiO_2_ layers, and the red and black lines indicate the position of the 3 nm-thick GdFeCo layers. The red ones denote the position of the remagnetized layers, and the black ones refer to the GdFeCo layers with untouched magnetization.

As it was mentioned above the femtosecond laser pulse propagating through GdFeCo layer performs ultrafast heating of the spin sublattices. The heating of a certain GdFeCo layer is determined by the energy of the laser pulse, i.e. |E|^2^, in that layer. The spatial distribution of |E|^2^ in the chirped MPC at the chosen operating wavelengths is given in Fig. 3. The value of |E|^2^ is given normalized to its maximum value at the certain wavelength. The spatial distributions of |E|^2^ as well as the spectral distribution in Fig. 2 have been numerically calculated by means of the rigorous coupled-wave analysis (RCWA)^39,40^. The choice of the method is due to the fact that it is suitable for the numerical analysis of both types of the GdFeCo layers addressed here, smooth and patterned layers.

The yellow areas in Fig. 3 correspond to TiO2 layers, the blue regions refer to SiO2 layers, and the red lines indicate the position of the 3 nm-thick GdFeCo layers. The positions of GdFeCo layers are chosen to coincide with the maximum value of |E|^2^ at each of four operating wavelengths, that provides the highest possible energy concentration inside the magnetic layer and its proper ultrafast heating as well.

The first GdFeCo layer is placed 19 nm in depth of the upper TiO_2_ layer and gets highest optical energy at λ = 644 nm (Fig. 3a). At that wavelength the EM field in the first GdFeCo layer $\left( {\left| E \right|_1^2} \right)$ exceeds the field in the other layers. In particular, the EM field in the second GdFeCo layer is $\left| E \right|_2^2 = 0.63\;\left| E \right|_1^2$, and the EM field in the third and fourth GdFeCo layers does not exceed 10% of $\left| E \right|_1^2$. Therefore, operating at the wavelength of 644 nm one can guarantee that light is mostly localized in the first magnetic layer and the magnetization switching takes place only in this layer. As a result, the first GdFeCo layer becomes ‘turned on’ while the rest magnetic layers remain untouched.

On the other hand, the second GdFeCo layer is put 40 nm inside the second TiO_2_ layer which ensures maximum concentration of the optical energy in this layer at λ = 686 nm. The third and fourth layers of GdFeCo are buried inside the corresponding TiO_2_ layer 21 nm and 83 nm in depth, respectively.

**Figure 4:**
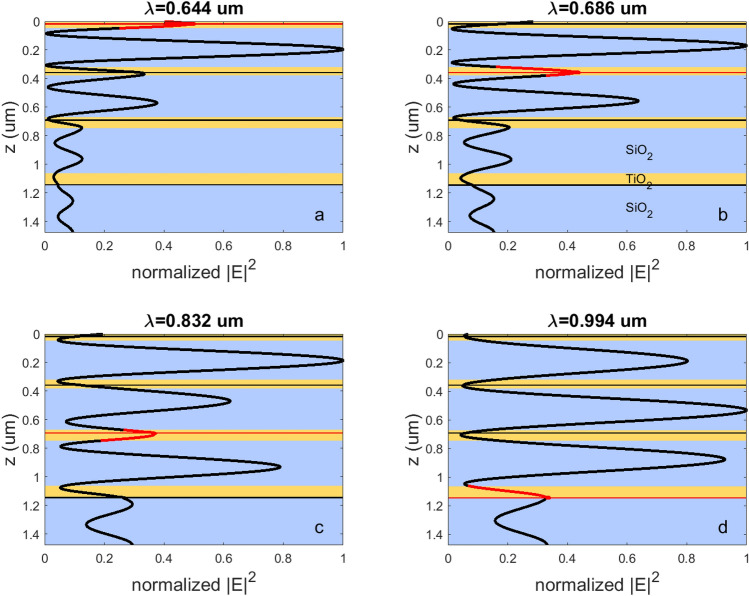
The spatial distribution of the normalized |E|^2^ in the chirped MPC with the patterned GdFeCo layer at the ‘operating’ wavelengths of (**a**) 644 nm, (**b**) 686 nm, (**c**) 832 nm, (**d**) 994 nm. Red sections of the curve show the magnitude of the normalized |E|^2^ inside the addressed GdFeCo layer and nearby. As in the Fig. 3 the yellow areas correspond to TiO_2_ layers, the blue regions refer to SiO_2_ layers, and the red and black lines indicate the position of the 9 nm-thick patterned GdFeCo layers. The red ones denote the position of the remagnetized layers, and the black ones refer to the GdFeCo layers with untouched magnetization.

Similarly, the spatial distribution of the normalized field |E|^2^ in the layers of the chirped MPC structure has been calculated for the case of patterned GdFeCo layers (Fig. 4). One can see that the same operating wavelengths provide that the intensity of light inside the targeted GdFeCo patterned layer exceeds the intensity localized in all other GdFeCo patterned layer.

Tables 2 and 3 give the detailed information on the ratio of the field values in different magnetic layers at the operating wavelengths for two addressed configurations. Table 2 refers to the MPC with smooth 3 nm-thick GdFeCo layers, and Table 3 presents data on the MPC configuration with 9 nm-thick patterned GdFeCo layers.

**Table 2:** The relative values of the maximum of |E|^2^ in the magnetic layers at the operating wavelengths in case of the smooth 3 nm-thick GdFeCo layer.

Wavelength	Max|E|^2^ in 1^st^ GdFeCo layer	Max|E|^2^ in 2^nd^ GdFeCo layer	Max|E|^2^ in 3^d^ GdFeCo layer	Max|E|^2^ in 4^th^ GdFeCo layer	Total absorption (%)
λ = 0.644um	1	0.6286	0.0710	0.0866	75
λ = 0.686um	0.3519	1	0.2011	0.1812	52
λ = 0.832um	0.4040	0.5605	1	0.7235	43
λ = 0.994um	0.0982	0.0896	0.0824	1	9

**Table 3:** The relative values of the maximum of |E|^2^ in the magnetic layers at the operating wavelengths in the case of the patterned 9 nm-thick GdFeCo layer.

Wavelength	Max|E|^2^ in 1st GdFeCo layer	Max|E|^2^ in 2nd GdFeCo layer	Max|E|^2^ in 3rd GdFeCo layer	Max|E|^2^ in 4th GdFeCo layer	Total absorption (%)
λ = 0.644um	1	0.6530	0.0894	0.0916	74
λ = 0.686um	0.4686	1	0.2286	0.1831	62
λ = 0.832um	0.3274	0.3572	1	0.6932	48
λ = 0.994um	0.1642	0.1596	0.1497	1	12

The data in the Tables 2 and 3 is given in the relative units. It means that the mean EM field |E|^2^ in the selected layer for each of the operating wavelengths is taken as 1 and the mean EM field in all others layers is compared with the field in the selected layer. It allows to show clearly that both types of the proposed chirped MPC allow to obtain a necessary excess of the EM field in the chosen GdFeCo layer compared to other magnetic layers. That excess is not less than 30%. That dramatic difference of the EM field energy in different magnetic layers of the MPC makes it possible to provide rather different levels of heating in different GdFeCo layers. Consequently, the considered chirped MPC structure allows to record information into each of the layers independently by femtosecond pulses at one of four wavelengths.

## Magnetization probing of the chirped magnetophotonic crystal

Now we discuss a method to probe the magnetization state of the magnetic layers in the proposed chirped magnetophotonic structure. There are various complicated methods for depth-resolved magnetization probing, for instance, by means of X-rays^41^ etc. However, the method proposed here is based on the measurement of the Faraday effect^32–44^ at a single wavelength of laser diode and could be easily implemented in data reading devices. The chosen parameters of the structure provide not only the heating above the threshold in different layers at the different operating wavelengths, but also different Faraday rotation angles by the magnetic layers. This difference lies in a root of magnetization probing approach proposed earlier in Ref.^45^ for the two-layer structure, however its extension to the *N*-layered structure requires performance of the measurements at *N* different wavelengths that is not convenient. We show that by measuring the magnitude of the Faraday effect at a certain wavelength, it is possible to determine the magnetization of each of the layers of the chirped MPC. We consider the Faraday rotation that is a polarization rotation of a transmitted light, however, exactly the same approach can be implemented to the polar Kerr effect measurements of the polarization rotation of the reflected light.

**Figure 5:**
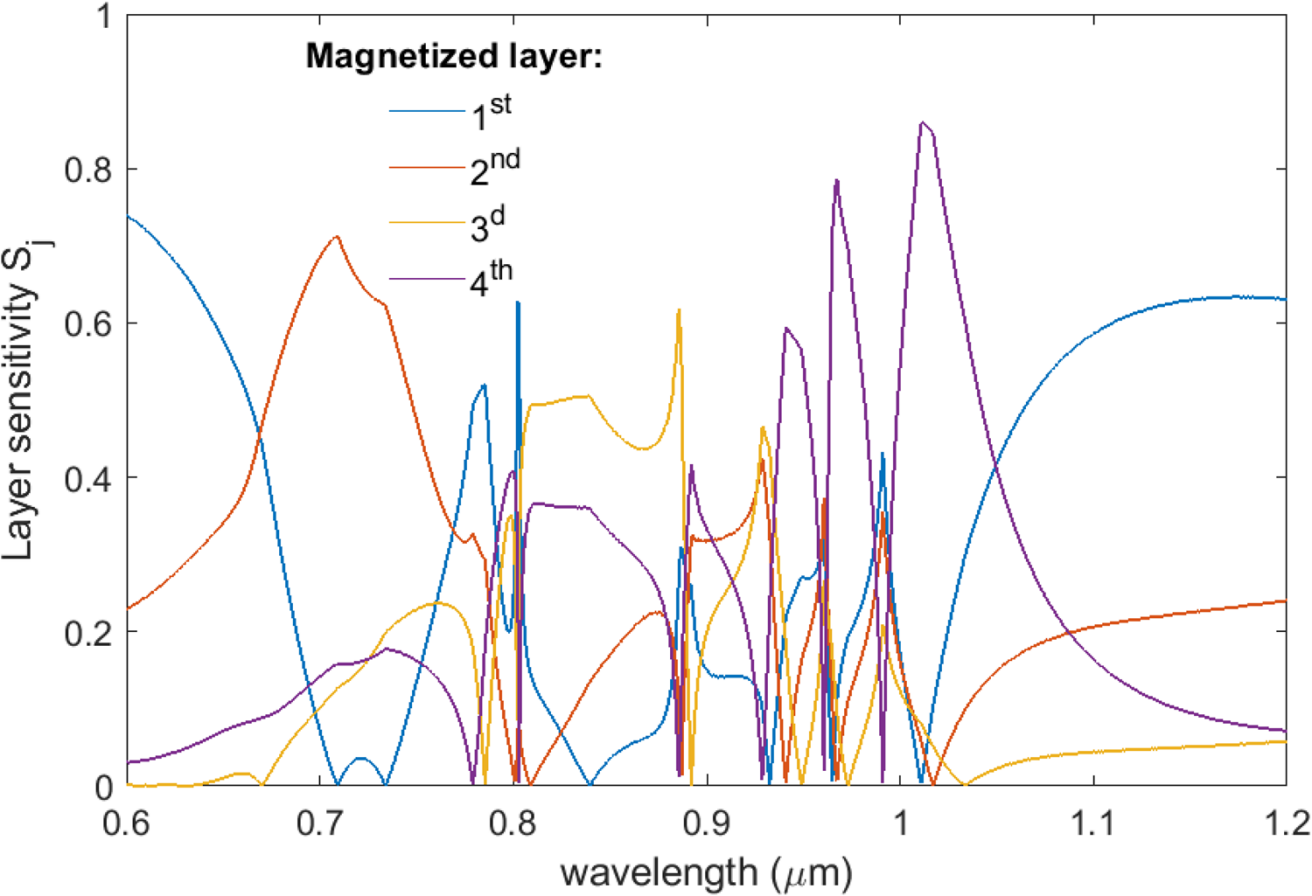
A layer sensitivity of the observed total Faraday rotation to the magnetization of a certain GdFeCo layer of the chirped MPC.

We determine the sensitivity of the observed total Faraday rotation to the magnetization of the *j*-th GdFeCo layer as follows

1 $$ {S_j} = \frac{{\left| {\Phi_j} \right|}}{{\sum\limits_{k = 1}^N {\left| {\Phi_k} \right|} }} $$

where ${\Phi_j}$ is a Faraday rotation angle of the linearly polarized light coming through the chirped MPC with magnetized *j*-th GdFeCo layer, *N* is a total number of the GdFeCo layers in the chirped MPC (here *N* = *4*). In the Fig. 5 the layer sensitivity ${S_j}\;\left( {j = \overline {1,4} } \right)$ versus the probing light wavelength is shown.

The Faraday effect of the chirped MPC depends strongly on the index number of the magnetized layer that means that the layer contributions to the observed total Faraday rotation are completely different. This fact allows for probing the magnetization of all four magnetic layers of the structure by measurement of the Faraday effect at a certain wavelength if 2^*N*^ magnetization states of the *N*-layered structure correspond to the 2^*N*^ different Faraday rotation angles. To provide such conditions, the operating probe wavelength should satisfy the following conditions. Firstly, the rotation angle of the polarization plane is noticeably different for each of the magnetic layers. Secondly, no combination of sums of Faraday angles from different magnetized layers is equal to another Faraday angle or other combination of sums. In other words, the Faraday rotation from one or several magnetized GdFeCo layers is different from the Faraday rotation provided by other magnetized layers of the chirped MPC. This method allows us to determine the magnetization state of each of the magnetic layers, that is, it will allow reading the information recorded in such 3D nanostructure.

This method could be understood using an example of toy model of the sensitivity ratios *S*_*1*_*:S*_*2*_*:S*_*3*_*:S*_*4*_ taken as 1:2:4:8 (so that ${S}_{1}=\frac{1}{15},{S}_{2}=\frac{2}{15},{S}_{3}=\frac{4}{15},{S}_{4}=\frac{8}{18}$ according to the normalization provided by the Eq. (1)) (see Table 4). Total Faraday rotation of the MPC is equal to $\Phi ={\Phi }_{0}({S}_{1}{m}_{1}+{S}_{2}{m}_{2}+{S}_{3}{m}_{3}+{S}_{4}{m}_{4})$, where ${m}_{j}=\pm 1$ denotes the magnetization direction of *j*-th layer, and ${\Phi }_{0}$ is the Faraday rotation angle corresponding to collinear magnetizations of the layers. Obviously, different 16 combinations of $\langle {m}_{1}{m}_{2}{m}_{3}{m}_{4}\rangle$ numbers correspond to 16 different values of $\Phi /{\Phi }_{0}$ which are in the range of $\frac{1}{15}(-15, -13,-11,\dots \mathrm{11,13}, 15)$. This is similar to the binary code of $\frac{\Phi }{{\Phi }_{0}}$ as $\langle {m}_{1}{m}_{2}{m}_{3}{m}_{4}\rangle$ (see Table 5).

**Table 4:** The layer sensitivity at three certain wavelengths, 0.98um, 1.064um, and 1.083um and in a toy model.

Wavelength	S_1_	S_2_	S_3_	S_4_
Toy model	0.0667	0.1333	0.2667	0.5333
λ = 0.980 μm	0.2344	0.1673	0.0593	0.5390
λ = 1.064 μm	0.4888	0.1750	0.0332	0.3030
λ = 1.083 μm	0.5512	0.1947	0.0405	0.2136

**Table 5:** The total Faraday rotation observed in the MPC with different magnetization states schematically shown by arrows at three wavelengths of 0.98um, 1.064um, and 1.083um and for a toy model.

λ μm	↑ ↑ ↑ ↑	↓ ↑ ↑ ↑	↑ ↓ ↑ ↑	↓ ↓ ↑ ↑	↑ ↑ ↓ ↑	↓ ↑ ↓ ↑	↑ ↓ ↓ ↑	↓ ↓ ↓ ↑	↑ ↑ ↑ ↓	↓ ↑ ↑ ↓	↑ ↓ ↑ ↓	↓ ↓ ↑ ↓	↑ ↑ ↓ ↓	↓ ↑ ↓ ↓	↑ ↓ ↓ ↓	↓ ↓ ↓ ↓
Toy model	− 1.00	− 0.87	− 0.73	− 0.6	− 0.47	− 0.33	− 0.2	− 0.07	0.07	0.2	0.33	0.47	0.6	0.73	0.87	1.00
0.980	− 1.00	− 0.53	− 0.66	− 0.20	− 0.88	− 0.41	− 0.55	− 0.07	0.07	0.55	0.41	0.88	0.20	0.66	0.53	1.00
1.064	− 1.00	− 0.02	− 0.65	0.32	− 0.93	0.04	− 0.58	0.39	− 0.39	0.58	− 0.04	0.93	− 0.32	0.65	0.02	1.00
1.083	− 1.00	0.10	− 0.61	0.49	− 0.92	0.18	− 0.53	0.57	− 0.57	0.53	− 0.18	0.92	− 0.49	0.61	− 0.1	1.00

Let us show how this method could be implemented in the considered MPC structure. The most convenient way, of course, is to operate at the wavelengths of the laser diodes. In this case there is no need in the wavelength-resolved measurements, but one can probe the magnetization of the GdFeCo layers exploring the Faraday effect at a certain wavelength. For example, the Nd laser diodes with the output wavelength of 1.064um are widespread and available with various output powers. The analysis given below shows that the magnetization state can be defined uniquely from the value of the Faraday rotation angle measured at a fixed wavelength.

In the Table 4 the layer sensitivities ${S_j}\;\left( {j = \overline {1,4} } \right)$ are given for three wavelengths corresponding to the output wavelength of the industrial laser diodes, 0.980um, 1.064um, and 1.083um. For a comparison, the values corresponding to the toy model in the example mentioned above are also provided.

In order to unambiguously probe the magnetization of a particular magnetic layer of the structure, it is necessary to ensure that two conditions are met. First, the Faraday rotation experienced by the probing light is different in case of magnetization of different GdFeCo layers. Second, no combination of the sums of values of Faraday rotations in different layers gives a magnitude of Faraday rotation of the plane of polarization in other layers. Analyzing the data given in Table 4 one can make sure that the first condition is met, since for any wavelength the sensitivity to magnetization of different layers is noticeably different. The validity of the second condition is discussed in Supplementary S3.

From analysis of the Table 5 one can made sure that the layer sensitivity values have a difference between themselves by at least 2% at the wavelengths of 0.980um, 1.064um, and 1.083um. This makes it possible to check that all of the possible 16 magnetization states provide the different Faraday rotation angles. Consequently, the proposed method of magnetization probing gives unambiguous results in the designed chirped MPC structure at any of these frequencies.

## Conclusions

To sum up, a design of the MPC structure providing a selective magnetization switching in the different magnetic layers at different frequencies is proposed. The MPC contains the thin layers of GdFeCo as magnetic counterparts. By illuminating the MPC by femtosecond laser pulses at the certain frequencies we create the conditions for the highest concentration of the electromagnetic field in the necessary layer of the structure while in the other GdFeCo layers the intensity is at least 1.5 times smaller. Thus, one can achieve selective all-optical magnetization reversal in a single layer of the multilayered stack determined by the laser frequency without any impact on the other layers. The magnetic layers of the MPC are separated from each other by the non-magnetic layers, and can be patterned, so, they can serve as the unit cells for information storage. We also provide a mechanism of single-wavelength reading of the information stored in such a multilayered stack. The approach was demonstrated for the 4 layers of SiO_2_, TiO_2_ and GdFeCo for the sake of simplicity, however it could be extended to the larger number of layers by the proper design of the chirped photonic crystal.

The reported study was funded by RFBR according to the research project no. 18-32-20225 mol_a_ved. Study of the selective sensing of the magnetization state of the photonic crystal with multiple magnetic layers was supported by Russian Science Foundation, Grant No. 19-72-10139.

## Supplementary Information

Supplementary Information

## References

[1] Kirilyuk A, Kimel AV, Rasing T (2010). Ultrafast optical manipulation of magnetic order. Rev. Mod. Phys..

[2] Kimel AV, Kirilyuk A, Usachev PA, Pisarev RV, Balbashov AM, Rasing T (2005). Ultrafast non-thermal control of magnetization by instantaneous photomagnetic pulses. Nature.

[3] Kimel AV, Kirilyuk A, Tsvetkov A, Pisarev RV, Rasing T (2004). Laser-induced ultrafast spin reorientation in the antiferromagnet TmFeO3. Nature.

[4] Kalashnikova AM, Kimel AV, Pisarev RV (2015). Ultrafast opto-magnetism. Phys. Usp..

[5] Bigot JY, Vomir M, Beaurepaire E (2009). Coherent ultrafast magnetism induced by femtosecond laser pulses. Nat. Phys..

[6] Boeglin C (2010). Distinguishing the ultrafast dynamics of spin and orbital moments in solids. Nature.

[7] Bigot J-Y (2013). Down to the nanometer scale. Nat. Mater..

[8] Radu I (2011). Transient ferromagnetic-like state mediating ultrafast reversal of antiferromagnetically coupled spins. Nature.

[9] Savochkin IV, Jäckl M, Belotelov VI, Akimov IA, Kozhaev MA, Sylgacheva DA (2017). Generation of spin waves by a train of fs-laser pulses: A novel approach for tuning magnon wavelength. Sci. Rep..

[10] Chernov AI, Kozhaev MA, Ignatyeva DO, Beginin EN, Sadovnikov AV, Voronov AA, Karki D, Levy M, Belotelov VI (2020). All-dielectric nanophotonics enables tunable excitation of the exchange spin waves. Nano Lett..

[11] Ostler TA (2012). Ultrafast heating as a sufficient stimulus for magnetization reversal in a ferrimagnet. Nat. Comms..

[12] Stanciu CD, Hansteen F, Kimel AV, Kirilyuk A, Tsukamoto A, Itoh A, Rasing T (2007). All-optical magnetic recording with circularly polarized light. Phys. Rev. Lett..

[13] Atxitia U, Ostler TA (2018). Ultrafast double magnetization switching in GdFeCo with two picosecond-delayed femtosecond pump pulses. Appl. Phys. Lett..

[14] Mentink JH (2012). Ultrafast spin dynamics in multisublattice magnets. Phys. Rev. Lett..

[15] Koopmans B (2010). Explaining the paradoxical diversity of ultrafast laser-induced demagnetization. Nat. Mater..

[16] Kirilyuk A, Kimel AV, Rasing T (2013). Laser-induced magnetization dynamics and reversal in ferrimagnetic alloys. Rep. Prog. Phys..

[17] Khorsand AR, Savoini M, Kirilyuk A, Kimel AV, Tsukamoto A, Itoh A, Rasing T (2012). Role of magnetic circular dichroism in all-optical magnetic recording. Phys. Rev. Lett..

[18] Vahaplar K (2012). All-optical magnetization reversal by circularly polarized laser pulses: Experiment and multiscale modelling. Phys. Rev. B.

[19] Davies CS, Janssen T, Mentink JH, Tsukamoto A, Kimel AV, van der Meer AFG (2020). Pathways for single-shot all-optical switching of magnetization in ferrimagnets. Phys. Rev. Appl..

[20] Steil D, Alebrand S, Hassdenteufel A, Cinchetti M, Aeschlimann M (2011). All-optical magnetization recording by tailoring optical excitation parameters. Phys. Rev. B.

[21] Zhang X, Rui G, Xu Y, Zhang F, Du Y, Lin X (2020). Fully controllable three-dimensional light-induced longitudinal magnetization using a single objective lens. Opt. Lett..

[22] Im S-J, Ri C-S, Ho K-S, Herrmann J (2017). Third-order nonlinearity by the inverse Faraday effect in planar magnetoplasmonic structures. Phys. Rev. B.

[23] Im S-J, Pea J-S, Ri C-S, Ho K-S (2019). Herrmann, All-optical magnetization switching by counterpropagataion or two-frequency pulses using the plasmon-induced inverse Faraday effect in magnetoplasmonic structures. Phys. Rev. B.

[24] Ignatyeva DO, Davies CS, Sylgacheva DA, Tsukamoto A, Yoshikawa H, Kapralov PO, Kirilyuk A, Belotelov VI, Kimel AV (2019). Plasmonic layer-selective all-optical switching of magnetization with nanometer resolution. Nat. Commun..

[25] Dutta A, Kildishev AV, Shalaev VM, Boltasseva A, Marinero EE (2017). Surface-plasmon opto-magnetic field enhancement for all-optical magnetization switching. Opt. Mater. Express.

[26] Borovkova OV, Hashim H, Kozhaev MA, Dagesyan SA, Chakravarty A, Levy M, Belotelov VI (2018). TMOKE as efficient tool for the magneto-optic analysis of ultra-thin magnetic films. Appl. Phys. Lett..

[27] Ignatyeva DO, Karki D, Voronov AA, Kozhaev MA, Krichevsky DM, Chernov AI, Levy M, Belotelov VI (2020). All-dielectric magnetic metasurface for advanced light control in dual polarizations combined with high-Q resonances. Nat. Commun..

[28] Voronov AA, Karki D, Ignatyeva DO, Kozhaev MA, Levy M, Belotelov VI (2020). Magneto-optics of subwavelength all-dielectric gratings. Opt. Express.

[29] Joannopoulos JD, Villeneuve PR, Fan S (1997). Photonic crystals: Putting a new twist on light. Nature.

[30] Chow E, Lin S, Johnson S, Villeneuve P, Joannopoulos J, Wendt JR, Vawter GA, Zubrzycki W, Hou H, Alleman A (2000). Three-dimensional control of light in a two-dimensional photonic crystal slab. Nature.

[31] Lyubchanskii IL, Dadoenkova NN, Lyubchanskii MI, Shapovalov EA, Rasing T (2003). Magnetic photonic crystals. J. Phys. D Appl. Phys..

[32] Inoue M (2006). Magnetophotonic crystals. J. Phys. D. Appl. Phys..

[33] Borovkova OV, Ignatyeva DO, Sekatskii SK, Karabchevsky A, Belotelov VI (2020). High-Q surface electromagnetic wave resonance excitation in magnetophotonic crystals for supersensitive detection of weak light absorption in the near-infrared. Photon. Res..

[34] Kozhaev MA, Chernov AI, Sylgacheva DA, Shaposhnikov AN, Prokopov AR, Berzhansky VN, Zvezdin AK, Belotelov VI (2018). Giant peak of the Inverse Faraday effect in the band gap of magnetophotonic microcavity. Sci. Rep..

[35] Rodríguez-de Marcos LV, Larruquert JI, Méndez JA, Aznárez JA (2016). Self-consistent optical constants of SiO_2_ and Ta_2_O_5_ films. Opt. Mater. Express.

[36] Sarkar S, Gupta V, Kumar M, Schubert J, Probst PT, Joseph J, König TAF (2019). Hybridized guided-mode resonances via colloidal plasmonic self-assembled grating. ACS Appl. Mater. Interfaces.

[37] Siefke T, Kroker S, Pfeiffer K, Puffky O, Dietrich K, Franta D, Ohlídal I, Szeghalmi A, Kley E-B, Tünnermann A (2016). Materials pushing the application limits of wire grid polarizers further into the deep ultraviolet spectral range. Adv. Opt. Mater..

[38] DeVore JR (1951). Refractive indices of rutile and sphalerite. JOSA.

[39] Moharam MG, Grann EB, Pommet DA, Gaylord TK (1995). Formulation for stable and efficient implementation of the rigorous coupled-wave analysis of binary gratings. J. Opt. Soc. Am. A.

[40] Li L (2003). Fourier modal method for crossed anisotropic gratings with arbitrary permittivity and permeability tensors. J. Opt. A.

[41] Zvezdin, A., Kotov, V. Modern magnetooptics and magneto-optical materials. *IOP Bristol*. (1997).

[42] Fischer P, Fadley CS (2012). Probing nanoscale behavior of magnetic materials with soft X-ray spectromicroscopy. Nanotechnol. Rev..

[43] Vinogradov AP, Inoue M, Levy M, Baryshev A (2013). Enhancement of the Faraday and Other Magneto-Optical Effects in Magnetophotonic Crystals. Magnetophotonics.

[44] Levy M, Borovkova OV, Sheidler C, Blasiola B, Karki D, Jomard F, Kozhaev MA, Popova E, Keller N, Belotelov VI (2019). Faraday rotation in iron garnet films beyond elemental substitutions. Optica.

[45] Tsema Y, Savoini M, Tsukamoto A, Kimel AV, Kirilyuk A, Rasing T (2016). Layer-sensitive magneto-optical spectroscopic study of magnetization dynamics in multilayered RE-TM structures. Appl. Phys. Lett..

