# Supplementary material for: Layer-selective magnetization switching in the chirped photonic crystal with GdFeCo
Source: Sci Rep. 2021 Jan 26;11:2239. doi: 10.1038/s41598-021-81887-0 (PMC7838275; doi:10.1038/s41598-021-81887-0)
Supplement: Supplementary file 1 — Supplementary Information [file 41598_2021_81887_MOESM1_ESM.pdf]

Supplementary to

'Layer-Selective Magnetization Switching in the Chirped Photonic Crystal with GdFeCo'

by O.V. Borovkova, D.O. Ignatyeva, and V.I. Belotelov

Supplementary S1.

The dielectric layers, SiO_2 and TiO_2, are considered to be lossless. The absorption is taken into account just inside the magnetic layers of GdFeCo. This assumption is valid due to the fact that the imaginary part of dielectric permittivity, ε", is 10-20 for GdFeCo in the addressed spectral range [17] as soon as it is about 10^{-3} in SiO_2 layer [36] and even less, 10^{-9}, in TiO_2 layers [37, 38]. The absorption in a material is proportional to the product of $\varepsilon''|E|^2$, where ε" is imaginary part of the layer permittivity. Although the energy of light concentrated in SiO_2 layers is one order greater than in TiO_2 and GdFeCo layers, the significant difference between the values of ε" for dielectric layers and magnetic material ensure that the absorption occurs just in the magnetic layers of the chirped MPC.

Supplementary S2.

The designed chirped MPC structures are rather tolerant to the various fabrication inaccuracies. We analyzed how the field distribution and its ratios in different GdFeCo layers depend on the variations of the magnetic layer thickness h_{GdFeCo}. In Fig. S1 it is shown how the maximum intensity of the field E in the smooth magnetic layers depends on the index number and its thickness at four operating wavelengths. One can see that when the thickness of the magnetic layers is less than 6nm the field intensity in the chosen layer exceeds the intensity in the other layers. Therefore, even in case of the small variations of the GdFeCo layers due to the fabrication inaccuracy the proposed design of the MPC provides to switch the magnetization in the targeted magnetic layer.

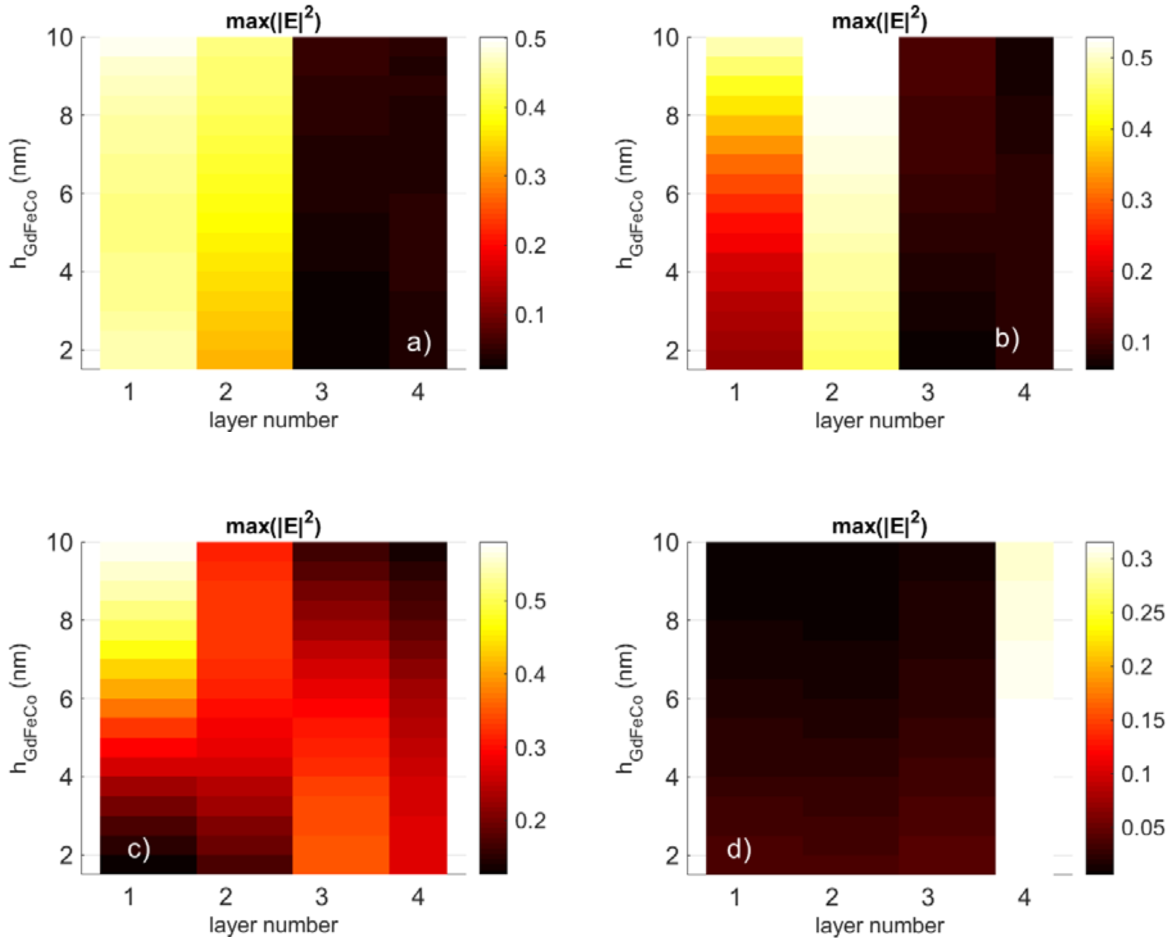

Figure S1. The maximum normalized $|E|^2$ in the chirped MPC with smooth GdFeCo layers versus layer number and magnetic layer thickness at the 'operating' wavelengths of (a) 644 nm, (b) 686 nm, (c) 832 nm, (d) 994 nm.

Supplementary S3.

In order to make sure that the second condition discussed in Section IV is satisfied, we will perform the following procedure. First of all, we sort the original sets of $S_j\left(j=\overline{1,4}\right)$ so that the resulting set $f_j\left(j=\overline{1,4}\right)$ is the ascending sequence ($f_1 < f_2 < f_3 < f_4$). Further, we compose various combinations of the sums $f_1 + f_2$, $f_2 + f_3$ etc. and compare them with the other elements of the sequences $f_j\left(j=\overline{1,4}\right)$. For instance, the ratio $\left(f_1 + f_2 - f_3\right)/f_3$ shows how the sum $f_1 + f_2$

differs from the value f_3. In particular, at the wavelength of 0.980um the sum $f_1 + f_2$ differs from f_3 by 3.3%. Similar ratios of various combinations of variables $f_j \left(j = \overline{1,4} \right)$ are given in Table S2.

Table S2. The relative difference of the layer sensitivity at three certain wavelengths, 0.98um, 1.064um, and 1.083um.

	λ=0.980um	λ=1.064um	λ=1.083um
$\frac{f_1 + f_2 - f_3}{f_3}$	3.3%	31.3%	10.1%
$\frac{f_1 + f_2 - f_4}{f_4}$	58%	57.4%	57.3%
$\frac{f_2 + f_3 - f_4}{f_4}$	25.5%	2.2%	26%
$\frac{f_1 + f_3 - f_4}{f_4}$	45.5%	31.2%	54%
$\frac{f_1 + f_2 + f_3 - f_4}{f_4}$	14.5%	4.6%	18.6%
$\frac{f_1 + f_4 - f_2 - f_3}{f_2 + f_3}$	49%	9.2%	45%

This makes it possible to check that no combination of the Faraday rotation in some layers will be equal to the combination of the Faraday rotations in the other magnetic layers.

Therefore, for the considered wavelengths, the constrained restrictions are fulfilled.
